# An infant with congenital heart defects and proteinuria: a case report

**DOI:** 10.1186/s12887-022-03705-4

**Published:** 2022-11-04

**Authors:** Dandan Liu, Yafeng Wang

**Affiliations:** 1 Department of Nephrology and Rheumatology, Department of Electrocardiogram, Children’s Hospital Affiliated to Zhengzhou University, Henan Children’s Hospital, Zhengzhou Children’s Hospital, Zhengzhou, 450018 China; 2 Department of Hematology and Oncology, Henan Provincial Key Laboratory of Children’s Genetics and Metabolic Diseases, Children’s Hospital Affiliated to Zhengzhou University, Henan Children’s Hospital, Zhengzhou Children’s Hospital, No.33 Longhuwaihuan East Road, Zhengzhou, 450018 China

**Keywords:** Branchio-Oto-Renal syndrome, Infants, Congenital heart defects, Proteinuria, Genetic sequencing analysis, Case report

## Abstract

**Background:**

Branchio-Oto-Renal (BOR) Syndrome is a rare autosomal disorder with a wide variety of clinical manifestations and a high degree of heterogeneity. Typical clinical manifestations of BOR syndrome include deafness, preauricular fistula, abnormal gill slits, and renal malformations. However, atypical phenotypes such as congenital hip dysplasia, congenital heart anomaly or facial nerve paresis are rare in BOR syndrome, and this might be easily misdiagnosed with other congenital disorders.

**Case presentation:**

We report a 5-month-old boy of BOR syndrome with "congenital heart defects and proteinuria" as clinical features. Initially, as this case mainly presented with symptoms of recurrent respiratory infections and was found to be with congenital heart disease and proteinuria at the local hospital, but he only was diagnosed with congenital heart disease combined with pulmonary infection and anti-infective and supportive treatment was given. Subsequently, during the physical examination at our hospital, left side preauricular pit and branchial fistulae on the right neck were found. Subsequent evaluation of auditory brainstem response and distortion product otoacoustic emission were revealed sensorineural hearing impairment. Results of renal ultrasonography showed small kidneys. Genetic analysis revealed a microdeletion at chromosome 8q13.2-q13.3 encompassing EYA1 gene, this patient was finally diagnosed with BOR syndrome. Then, this patient received transcatheter patent ductus arteriosus closure and hearing aid treatment. Proteinuria, renal function and hearing ability are monitoring by nephrologist and otologist. The patient is currently being followed up until 3 months after discharge and his condition is stable.

**Conclusion:**

Careful physical examination, detailed history and the implementation of diagnostic laboratory tests can reduce the incidence of misdiagnosis. Genetic sequencing analysis of patients is a key guide to the differential diagnosis of BOR syndrome.

## Background

In 1976, Branchio-Oto-Renal (BOR) syndrome was first described by Melnick et al. [1], which is a rare autosomal disorder with a wide variety of clinical manifestations and a high degree of heterogeneity, and the phenotype and severity can vary widely among patients in the same family [2]. Typical clinical manifestations of BOR syndrome include deafness, preauricular fistula, abnormal gill slits, and renal malformations [2, 3]. However, when patients have a combination of multisystem congenital malformations or organ developmental disorders, the misdiagnosis is likely be made. With the development of genetics in recent years, the causative genes of BOR syndrome in several patients have been recognized, such as *EYA1* which is most commonly associated causative gene [4]. The chromosomal region, microdeletions or microduplications within which can also lead to the BOR syndrome [2]. In the article, we report a case of BOR syndrome with "congenital heart defects and proteinuria" as clinical features.

## Case presentation

A 5-month-old boy with a history of recurrent respiratory infection was presented during the past 1 month. He had a recurrent cough. He was presented to a local hospital and was presumptively diagnosed with lung infection, congenital heart defect and proteinuria. After antibiotics treatment and supportive care, he was discharged home. Due to the persistent positive proteinuria, this boy was admitted to our hospital for further treatment. He was born at the 39th week gestation, by cesarean section. The parents denied any family history of congenital defects or genetic diseases. During pregnancy, the imaging examination of his mother of the patient showed no abnormalities. His mother denied the history of exposure to radiation, toxic chemicals, drugs that are toxic to the fetus, contaminated water and air during pregnancy.

**Fig. 1:**
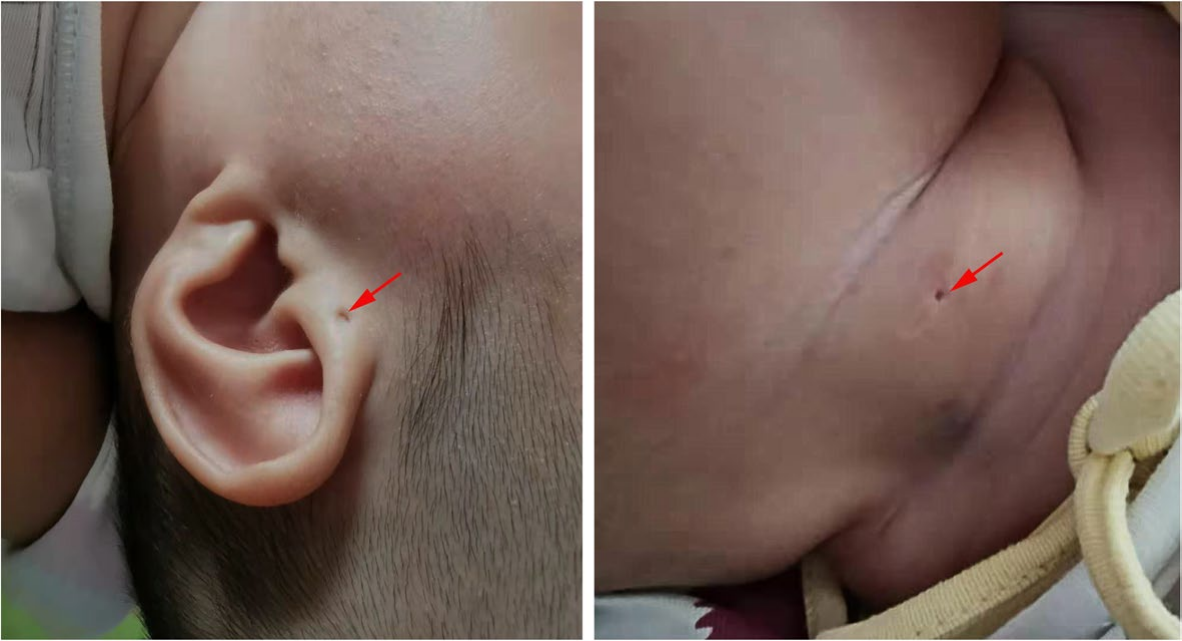
Photograph of infant’s preauricular pit (left panel) and branchial fistulae (right panel)

On examination, the patient was afebrile without rash. He had left side preauricular pit and branchial fistulae on the right neck (Fig. 1). The mitral first sound was a little loud, and the second sound was normal. At the pulmonic area, a continuous, machinery type of murmur was heard, grade II-III.

Laboratory evaluation revealed normal results for complete blood cell count, C-reactive protein, levels of electrolytes, creatinine, and liver enzymes. Urine dipstick protein value was 1 + (reference range, negative), and a 24-h urine collection revealed 180 mg of albumin (reference range, < 150 mg/24 h). An echocardiography showed the presence of a patent ductus arteriosus (PDA) and patent foramen ovale. Computed tomography (CT) of the chest showed pleural thickening.

The concurrent presence of preauricular recess, bronchial fistula and proteinuria in this patient triggered consideration of the diagnosis of BOR syndrome. However, further testing of this patient's hearing, kidneys was required, and whether this infant have mutated genes was also crucial to the correct diagnosis. In addition, this patient also has an abnormal heart development and genetic analysis may provide additional diagnostic evidence to distinguish BOR syndrome from other disorders.

**Fig. 2:**
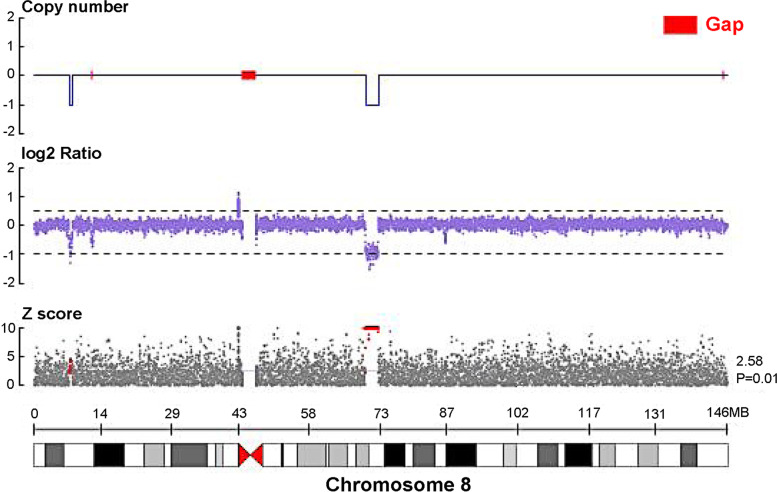
Patient’s genetic analysis revealed a microdeletion at chromosome 8q13.2-q13.3 encompassing *EYA1* gene

Subsequent evaluation of auditory brainstem response and distortion product otoacoustic emission were revealed sensorineural hearing impairment. Results of renal ultrasonography showed small kidneys. The kidneys were measured 4.4 cm and 4.1 cm in length (normal for the patient’s age, 5.7 cm). Genetic analysis revealed a microdeletion at chromosome 8q13.2-q13.3 encompassing *EYA1* gene (Fig. 2). Thus, combining his clinical presentation, laboratory test results and genetic sequencing analysis, this patient was finally diagnosed with BOR syndrome.

For treatment, this infant received transcatheter PDA closure and hearing aid treatment. Proteinuria, renal function and hearing ability are monitoring by nephrologist and otologist every month. The patient is currently being followed up until 3 months after discharge and his condition is stable.

## Discussion and conclusions

BOR syndrome is an autosomal dominant disorder characterized by branchial, ear and renal abnormalities. It occurs in approximately 2% of profoundly deaf children, with the prevalence of 1 in 40000 [5]. The common phenotypes of BOR syndrome include hearing loss, preauricular pits, auricular malformation, branchial anomalies, and renal anomalies [6]. Hearing loss is the most common phenotypic manifestation, the severity may range from mild to profound in degree [7, 8]. Renal anomalies also vary in individuals from mild hypoplasia to agenesis [7]. Branchial anomalies normally include the branchial cleft sinus tract and branchial cleft cyst, and sometimes a pedunculated papule can be observed [2, 9]. Besides, congenital hip dysplasia, congenital heart anomaly, facial nerve paresis, and other findings are rare and have been reported in BOR syndrome [2, 7]. The major criteria include second branchial arch anomalies, hearing impairment, preauricular pits, and renal anomalies. The minor criteria include external, middle, or internal ear anomalies, preauricular tags, facial asymmetry and palate abnormalities. The clinical diagnosis of BOR syndrome can be made to patients without a family history who have any 3 major criteria or 2 major criteria and 2 minor criteria, or patients with a family history who have 1 major criteria [6]. Because of the high degree of variability in the clinical presentation of BOR syndrome, a detailed history and careful physical examination are essential to make a correct diagnosis.

*EYA1* (OMIM 601,653) *SIX1* (OMIM 601,205) and *SIX5* (OMIM 600,963) gene have been reported as the common causative gene of BOR syndrome [10, 11]. In European and American populations, about 40% of BOR syndrome patients had mutations in *EYA1* gene, of which 80% were point mutations or small fragment deletion and 20% were large fragment deletion [6, 12]. In contrast to the western population with large cohort studies, limited information is available regarding to *EYA1* mutations of BOR syndrome in the East Asian population, such as approximately 71% *EYA1* mutations was identified in a Korean cohort [13]. The relationship between different mutation of *EYA1* gene and clinical phenotype of BOR syndrome is still unclear. In the same consanguineous family, different family members with BOR syndrome may occur different symptoms. For example, intellectual disability is uncommon in patients with distinctive BOR syndrome, however, patients presenting with intellectual disability may have other syndromes or chromosomal microdeletions in *EYA1* [12]. Patients with a microdeletion at 8q13.2-q13.3 encompassing *EYA1* have been described to be associated with BOR syndrome, as well as non-relevant BOR syndrome abnormalities such as cardiac defects, toe hypoplasia, and abnormal clavicles [3, 12]. Cayler cardiofacial syndrome is characterized by asymmetric crying face and congenital heart defects, which is associated with the T468fsX482 mutation in *EYA1* [14]. Facial asymmetry and congenital heart defects are rare anomalies in BOR syndrome [7], thus, genetic analysis is crucial for the differential diagnosis of patients with uncommon phenotypes of BOR syndrome.

Symptomatic treatments are the main strategies for patients with BOR syndrome. Infected auricular or branchial fistulae can be performed antibiotics or removed if they are recurrently infected. Hearing aid and surgical operations are effective for patients with different degrees of hearing loss. Renal symptoms can be treated surgically or renal replacement therapy, depends on the severity of the renal insufficiency [2]. For this patient, his quality of life improved after heart surgery and hearing aid treatment, however, he still needs outpatient follow-up of his kidney function and the severity of his proteinuria in order to receive timely and effective treatment.

In this article, we describe a case of BOR syndrome with typical symptoms and an atypical phenotype. When encountering a patient with suspected BOR syndrome, careful physical examination, detailed medical history and the implementation of diagnostic laboratory tests are required. Genetic sequencing analysis of patients with suspected BOR syndrome is not only diagnostically important, but is also a key guide to the differential diagnosis of BOR syndrome. The current treatment of BOR syndrome is mainly symptomatic support. Avoidance of consanguineous marriages and prenatal diagnosis and genetic counselling in families with clear causative genetic mutations can effectively reduce the number of infants born with BOR syndrome.

## Data Availability

The original contributions presented in the study are included in the article, further inquiries can be directed to the corresponding author.

## Abbreviations

BOR Syndrome: Branchio-Oto-Renal syndrome
PDA: Patent ductus arteriosus
CT: Computed tomography
OMIM: Online Mendelian Inheritance in Man
